# Lafora disease in miniature Wirehaired Dachshunds

**DOI:** 10.1371/journal.pone.0182024

**Published:** 2017-08-02

**Authors:** Lindsay Swain, Gill Key, Anna Tauro, Saija Ahonen, Peixiang Wang, Cameron Ackerley, Berge A. Minassian, Clare Rusbridge

**Affiliations:** 1 Fitzpatrick Referrals Orthopedics and Neurology, Halfway Lane, Eashing, Godalming, Surrey, United Kingdom; 2 Dachshund Breed Council, Wrington, North Somerset, United Kingdom; 3 Program in Genetics and Genome Biology, The Hospital for Sick Children, Toronto, Canada; 4 Department of Pathology and Laboratory Medicine, The Hospital for Sick Children, University of Toronto, Toronto, Canada; 5 Department of Pediatrics (Neurology), The Hospital for Sick Children, University of Toronto, Toronto, Canada; 6 School of Veterinary Medicine, Faculty of Health & Medical Sciences, University of Surrey, Guildford, Surrey, United Kingdom; Sant Joan de Déu Children's Hospital, SPAIN

## Abstract

Lafora disease (LD) is an autosomal recessive late onset, progressive myoclonic epilepsy with a high prevalence in the miniature Wirehaired Dachshund. The disease is due to a mutation in the *Epm2b* gene which results in intracellular accumulation of abnormal glycogen (Lafora bodies). Recent breed-wide testing suggests that the carrier plus affected rate may be as high as 20%. A characteristic feature of the disease is spontaneous and reflex myoclonus; however clinical signs and disease progression are not well described. A survey was submitted to owners of MWHD which were homozygous for *Epm2b* mutation (breed club testing program) or had late onset reflex myoclonus and clinical diagnosis of LD. There were 27 dogs (11 male; 16 female) for analysis after young mutation-positive dogs that had yet to develop disease were excluded. Average age of onset of clinical signs was 6.94 years (3.5–12). The most common initial presenting sign was reflex and spontaneous myoclonus (77.8%). Other presenting signs included hypnic myoclonus (51.9%) and generalized seizures (40.7%). Less common presenting signs include focal seizures, “jaw smacking”, “fly catching”, “panic attacks”, impaired vision, aggression and urinary incontinence. All these clinical signs may appear, and then increase in frequency and intensity over time. The myoclonus in particular becomes more severe and more refractory to treatment. Signs that developed later in the disease include dementia (51.9%), blindness (48.1%), aggression to people (25.9%) and dogs (33.3%), deafness (29.6%) and fecal (29.6%) and urinary (37.0%) incontinence as a result of loss of house training (disinhibited type behavior). Further prospective study is needed to further characterize the canine disease and to allow more specific therapeutic strategies and to tailor therapy as the disease progresses.

## Introduction

Lafora disease (LD) is an autosomal recessive myoclonic epilepsy caused by mutations in the *EPM2A* gene, encoding the glycogen phosphatase laforin, or the *EPM2B* gene coding the ubiquitin E3 ligase malin [1–3]. In humans, LD is arguably the severest adolescence-onset epilepsy [4]. Its early signs are myoclonus and generalized tonic-clonic seizures, along with transient blindness, visual hallucinations (often with frightening content) or photoconvulsions. As the disease progresses the severity, frequency and refractoriness of the seizures increase. Ataxia and emotional disturbances develop, followed by spasticity and dementia. A vegetative state in constant myoclonus precedes death, which occurs in status epilepticus approximately 10 years after onset [3, 5]. The mutations in human LD are numerous, spread in all regions of either gene, all leading to loss of function [2, 6].

**Fig 1:**
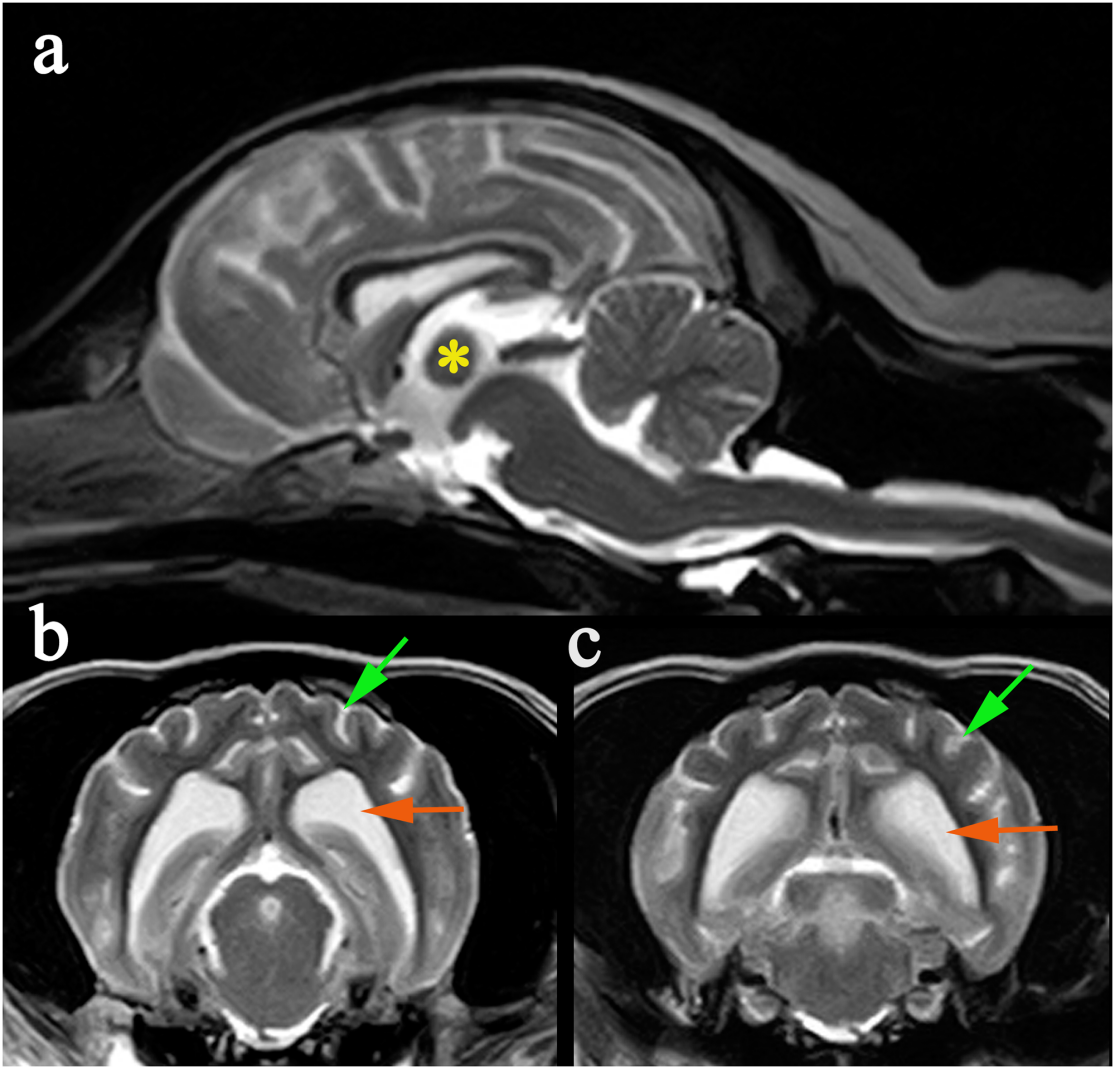
MRI changes in an 8 year old male neutered MWHD with advanced LD. (a) Mid-sagittal T2W image of the brain demonstrating atrophy of the intra-thalamic adhesion (*). (b) Transverse T2W at the level of temporal lobes demonstrating cortical atrophy with widening of the subarachnoid space (green arrow) and enlargement of the lateral ventricle (orange arrow). (c) Transverse T2W at level of occipital lobes demonstrating cortical atrophy with widening of the subarachnoid space (green arrow) and enlargement of the lateral ventricle (orange arrow).

In dogs, LD is also inherited as an autosomal recessive condition and is one of the most common recognized structural-metabolic epilepsies [7]. It is most frequent in Miniature Wirehaired Dachshunds (MWHD) [1, 8, 9], Bassett Hounds [1, 10] and Beagles [11, 12], and single cases have been reported in the Miniature and Standard Poodle [13], Pointer [14], and a Corgi [15]. LD has also been reported in other species including a Fennec fox [16], cows [17] and possibly a parakeet [18]. Clinical signs are similar to those of the human disease, including spontaneous and reflex myoclonus, hypnic jerks and generalized tonic clonic seizures [1]. Complete blood count, serum biochemistry, urinalysis and cerebrospinal fluid are normal [8, 9]. Electroencephalographic (EEG) measurements reveal bilateral synchronous polyspike-wave paroxysms and erratic myoclonus without EEG correlation [11]. Magnetic resonance imaging (MRI) may reveal generalized cortical atrophy with ventriculomegaly (Fig 1) [1, 9]. Needle electromyography (EMG) has been reported to show abnormal spontaneous activity of the muscles with positive sharp waves and fibrillation potential [8].

**Fig 2:**
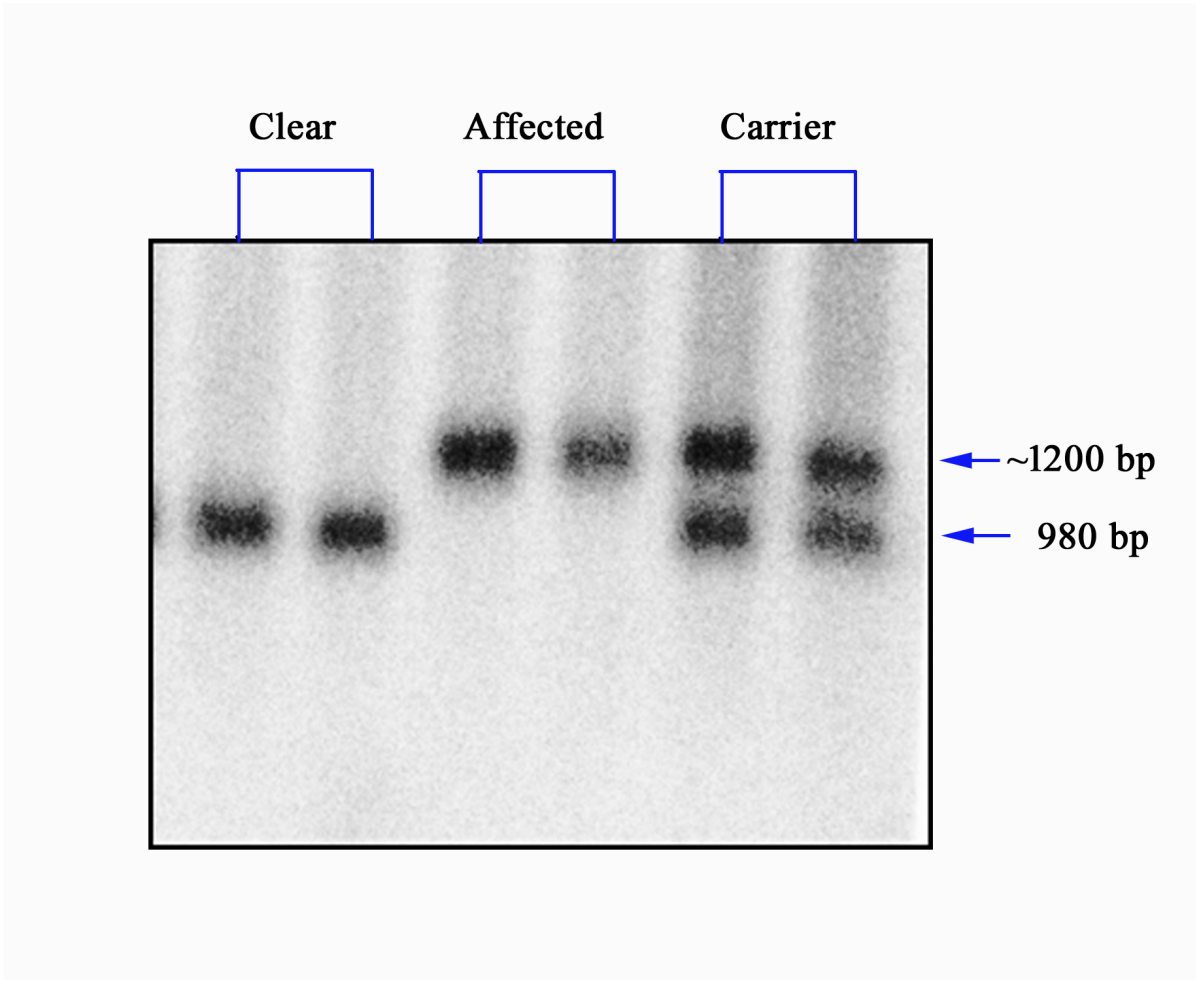
A representative of EPM2B Southern blot results. The DraIII-EcoR fragment of EPM2B gene in wild-type animal is 980 base pairs (bp), while the same fragment of EPM2B mutation allele in affected or Carrier animals is around 1200 bp.

To date, a single mutation has been found in canine LD, in all three of three breeds (MWHD, Bassett Hounds and Beagles) in which mutation testing has been performed [1, 12]. This mutation consists of a massive expansion of a dodecamer repeat sequence present exclusively in the canine (and generally canid) *Epm2b* gene, leading to near-absent expression and loss of function of the gene [1, 12]. Genetic testing is not routinely available because the massive dodecamer repeat expansion mutation makes PCR-based sequencing options unreliable for detecting the carrier state and consequently only a southern blot based test is offered by the University of Toronto and is an official DNA screening test recommended by the UK Kennel Club (Fig 2)

**Fig 3:**
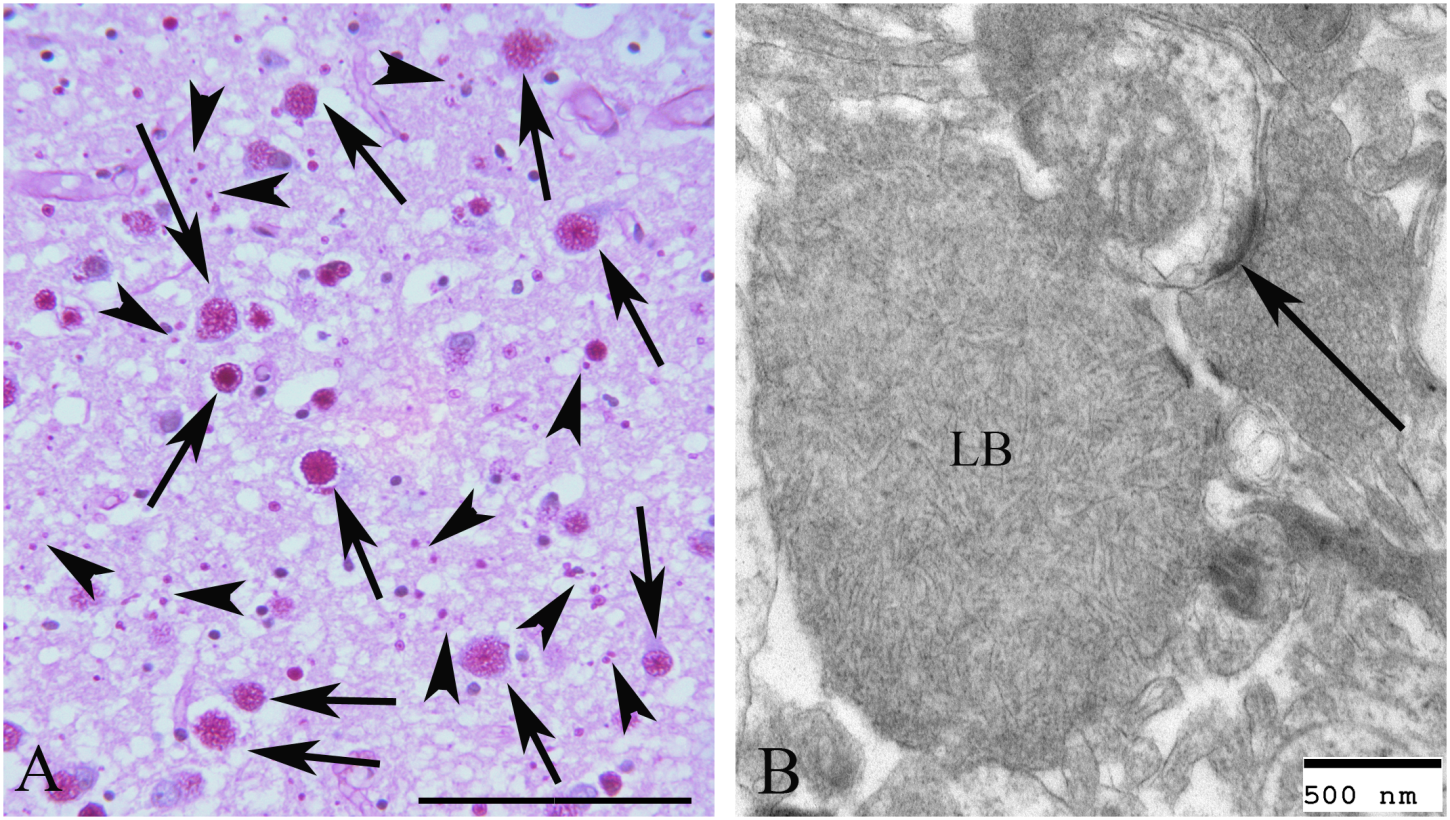
Lafora bodies in the frontal cortex of a MWHD. A. PAS diastase (PASD) treated section of frontal cortex from the brain of a MWHD with Lafora disease collected at autopsy. Note the numerous PASD positive Lafora Bodies (LB) (arrows) located in the perikaryon of many of the neurons. Much smaller LBs are located throughout (small arrows). Bar equals 50μm. B. Electron micrograph of a LB in the terminal cytoplasm of a dendrite. A synaptic density is seen (arrow). Bar equals 500nm.

The underlying pathology in LD is transformation of spherical and soluble glycogen to a more linear and insoluble glucan called polyglucosan. Gradual precipitation, aggregation, and accumulation of polyglucosans results in massive neurotoxic inclusions (Lafora bodies; LB), which drive the neurodegenerative epilepsy [3] (Fig 3).

Precisely how the laforin-malin partner enzymes regulate glycogen structure remains unclear [19, 20]. Notwithstanding, a shortcut to therapy was recently suggested. Glycogen synthase is the only enzyme that catalyzes formation of α1–4 interglucosidic linkages that generate glycogen (or polyglucosan) strands. Mere 50% reduction of glycogen synthase activity in LD mouse models through transgenic approaches was shown to eliminate LB and the neurological phenotype of the disease [5, 21–23]. As such, work is actively underway to identify glycogen synthase inhibitors towards a potential therapy for LD, human and canine. Clinical trials require detailed description of the clinical disease and its natural progression. This paper fills an important part of this void for canine LD.

## Ethics statement

This retrospective study was based on the analysis of responses to a confidential online survey which did not collect personal data and no aspect comes under the auspices of Animals (Scientific Procedures) Act 1986. Informed written consent to use data was obtained from survey participants. Ethics committee approval for this project was not sought because the decision to create and run the survey was made by the Lafora disease subcommittee (part of the Dachshund Breed Council) and ethics approval cannot be made retrospectively.

## Materials and methods

The Dachshund Breed Council is affiliated with the UK Kennel Club and represents the interests of sixteen UK Dachshund Breed Clubs and appoints Health and Welfare Sub-committees to develop policies and coordinate plans for Dachshund breed health improvement. One of these is the LD Subcommittee which constructed an online survey in 2012 with the aim of assessing the clinical signs and impact of LD [24, 25]. Common signs of disease were identified: myoclonic jerks (spontaneous and reflex, most commonly induced by light, sound or sudden movement in the visual field), hypnic myoclonus (twitch as falling asleep), seizures (both focal and tonic-clonic), ataxia, poor vision/blindness, deafness, dementia (defined as a behavior change suggesting disorientation and memory loss with or without altered sleep wake cycle and anxiety), aggression (toward people and other dogs), incontinence due to loss of house training i.e. disinhibited behavior (both fecal and urinary), jaw smacking and fly catching however the survey did not include data on how this disease progressed. Consequently, in 2013 the online survey was refined to include information about age of onset, progression of clinical signs, comorbidities, impact of disease and, if applicable, age of death (S1 Text) [26]. The confidential online survey ran between January 2013 and January 2014. Participants were invited if they had previously contacted the MWHD Lafora subcommittee about pets with clinical signs of LD and/or if they had participated in bi-annual bulk genetic testing for LD through the Dachshund Breed Council [25]. Participants gave their written consent for the clinical data to be used for research purposes and the raw data from the survey (S1 Table) was subsequently passed to this group with a request for analysis to provide information on disease progression and further knowledge of the disease.

Inclusion criteria for the analysis was a UK bred MWHD that had either positive genetic testing for LD or had showed pathognomonic signs of LD including reflex myoclonic jerks, had medical history available for review and died before genetic testing was available. Survey responses were excluded if the dogs were not UK bred MWHD or were mutation positive and not yet showing clinical signs. All dogs that tested negative for LD were also excluded.

## Results

### Signalment

Forty-one online responses were received. Fourteen dogs were immediately excluded from the study as they were not showing clinical signs at the time of the study; four of these dogs had tested positive for the LD disease mutation but did not yet show clinical signs (all these dogs were younger than the age of onset of canine LD). Of the remaining 27 dogs, 16 were confirmed homozygous for the LD mutation via genetic testing. The remaining 11 dogs were not tested as they died prior to the genetic test being available. All, however, had the classical clinical disease including spontaneous and reflex myoclonic jerks. The signalment results are illustrated in Table 1. 62.5% of the cohort was female. The mean age of onset of clinical signs was 6.94 years which correlated with the median age of onset of 7 years. The range of the onset of clinical signs was wide at 3.5 to 12 years.

**Table 1:** Signalment and age of onset of LD.

	Mutation-Positive	Gene test not performed	Combined
**Number of dogs**	16	11	27
**Mean age of onset (years)**	6.66	7.46	6.94
**Median age of onset (years)**	6.5	7.0	7.0
**Range of age of onset (years)**	3.5–9.0	6.0–12.0	3.5–12.0
**Male**	6 (37.5%)	5 (45.5%)	11 (40.7%)
**Female**	10 (62.5%)	6 (55.5%)	16 (59.2%)

Signalment and age of onset of first clinical signs in a cohort of data for 27 MWHD with confirmed and suspected LD.

### Clinical signs at presentation

Table 2 illustrates the distribution of fourteen common clinical signs at presentation in LD for both mutation-positive and untested cases. The most common sign for both were reflex and spontaneous myoclonus, hypnic myoclonus, generalized seizures, and jaw smacking. The remaining clinical signs at presentation were fly catching and panic attacks, focal seizures, aggression toward people, urinary incontinence as a result of loss of house training, poor vision or blindness, fecal incontinence, or dementia.

**Table 2:** Fourteen presenting clinical signs of LD.

Presenting Clinical Signs	Mutation-positive (number and percentage)	Gene test not performed (number and percentage)	Total (number and percentage)
**Reflex and spontaneous myoclonus**	12 (70.5%)	9 (81.8%)	21 (77.8%)
**Hypnic myoclonus**	8 (50.0%)	6 (54.5%)	14 (51.9%)
**Generalized tonic clonic seizures**	6 (37.5%)	5 (45.5%)	12 (40.7%)
**Jaw Smacking**	5 (31.5%)	4 (36.4%)	9 (33.3%)
**Fly Catching**	4 (25.0%)	2 (18.2%)	7 (22.2%)
**Panic Attacks**	3 (18.8%)	4 (36.4%)	7 (25.9%)
**Focal Seizures**	2 (12.5%)	5 (45.5%)	7 (25.9%)
**Aggression toward people**	1 (6.3%)	3 (27.3%)	4 (14.8%)
**Aggression toward dogs**	1 (6.3%)	3 (27.3%)	4 (14.8%)
**Poor vision/blindness**	1 (6.3%)	3 (27.3%)	4 (14.8%)
**Urinary Incontinence**	1 (6.3%)	2 (18.2%)	3 (11.1%)
**Fecal incontinence**	0 (0.0%)	1 (9.1%)	1 (3.7%)
**Dementia**	0 (0.0%)	1 (9.1%)	1 (3.7%)
**Deafness**	0 (0.0%)	0 (0.0%)	0 (0.0%)

Distribution of clinical signs in the LD affected and suspected dogs at presentation

### Early clinical signs (years 1–3 following onset)

In the gene test-positive group the most frequent new clinical sign that developed over the first 1–3 years following presentation was poor vision /blindness which was reported in over one third of the dogs. Other common signs were generalized tonic clonic seizures, hypnic myoclonus, panic attacks and dementia. Less frequent signs were jaw smacking, fly catching, focal seizures, aggression toward other dogs, fecal incontinence as a result of loss of house training, and deafness. The untested group had slightly different development of clinical signs, the most frequent being dementia, which was reported in two thirds of the cases, followed by focal seizures. Less frequent clinical signs are illustrated in Table 3

**Table 3:** Early clinical signs in LD.

Clinical Signs at 1–3 years of disease	Mutation-positive (number and percentage)	Gene test not performed (number and percentage)	Total (number and percentage)
**Reflex and spontaneous myoclonus**	13 (81.3%)	11 (100%)	24 (88.9%)
**Hypnic myoclonus**	11 (68.8%)	10 (90.9%)	21 (77.8%)
**Generalized tonic clonic seizures**	10 (62.5%)	9 (81.8%)	19 (70.4%)
**Jaw Smacking**	7 (43.8%)	5 (45.5%)	12 (44.4%)
**Fly Catching**	5 (31.3%)	4 (36.4%)	9 (33.3%)
**Panic Attacks**	6 (37.5%)	7 (63.6%)	13 (48.1%)
**Focal Seizures**	3 (18.8%)	9 (81.8%)	12 (44.4%)
**Aggression toward people**	1 (6.25%)	4 (36.4%)	5 (18.5%)
**Aggression toward dogs**	2 (12.5%)	3 (27.3%)	5 (18.5%)
**Poor vision/blindness**	6 (37.5%)	5 (45.5%)	11 (40.7%)
**Urinary Incontinence**	2 (12.5%)	4 (36.4%)	6 (22.2%)
**Fecal incontinence**	2 (12.5%)	3 (27.3%)	5 (18.5%)
**Dementia**	3 (18.8%)	8 (72.7%)	11 (40.7%)
**Deafness**	2 (12.5%)	3 (27.3%)	5 (18.5%)

Distribution of clinical signs in the LD affected and suspected dogs after 1–3 years of disease

### Late clinical signs (>3 years)

Late onset clinical signs (those developing 3 years or more after initial presentation) in the mutation-positive group were dementia followed by panic attacks, urinary incontinence, and deafness (Table 4). In the untested group the most common developing late clinical signs were those of aggression towards other dogs and urinary and fecal incontinence as a result of loss of house training (disinhibited).

**Table 4:** Late clinical signs in LD.

Clinical Signs at 3 or more years of disease	Mutation-positive (number and percentage)	Gene test not performed (number and percentage)	Total (number and percentage)
**Reflex and spontaneous myoclonus**	13 (81.3%)	11 (100%)	24 (88.9%)
**Hypnic myoclonus**	11 (68.8%)	10 (90.9%)	21 (77.8%)
**Generalized tonic clonic seizures**	10 (62.5%)	9 (81.8%)	19 (70.4%)
**Jaw Smacking**	7 (43.8%)	5 (45.5%)	12 (44.4%)
**Fly Catching**	5 (31.3%)	4 (36.4%)	9 (33.3%)
**Panic Attacks**	8 (50.0%)	7 (63.6%)	15 (55.6%)
**Focal Seizures**	3 (18.8%)	10 (90.9%)	13 (48.1%)
**Aggression toward people**	2 (12.5%)	5 (45.5%)	7 (25.9%)
**Aggression toward dogs**	2 (12.5%)	7 (63.6%)	9 (33.3%)
**Poor vision/blindness**	7 (43.8%)	6 (54.5%)	13 (48.1%)
**Urinary Incontinence**	4 (25.0%)	6 (54.5%)	10 (37.0%)
**Fecal incontinence**	3 (18.8%)	5 (45.5%)	8 (29.6%)
**Dementia**	6 (37.5%)	8 (72.7%)	14 (51.9%)
**Deafness**	4 (25.0%)	4 (36.4%)	8 (29.6%)

Distribution of clinical signs in the LD affected and suspected dogs after 3 years of disease

## Discussion

Our study showed that LD in the MWHD is a progressive myoclonic epilepsy similar to the disease process in humans. The disease is late onset with an average age of approximately 7 years at presentation. The cardinal features of the disease are: spontaneous myoclonus, reflex myoclonus (myoclonus in response to light, sounds and / or movement in visual field), hypnic jerks (jerking as falling asleep), generalized seizures, focal seizures, anxiety, impaired vision, and later cognitive decline to dementia, ataxia, deafness, and fecal and urinary incontinence as a result of loss of house training (disinhibited behavior). All these clinical signs appear, and then increase over time, in all dogs (S1 Movie). Myoclonus, in particular, becomes progressively more severe although anecdotally there is an initial response to levetiracetum. Owners described the (presumed) panic attacks as particularly distressing and from the description [24] we hypothesize that the dogs may be experiencing frightening visual hallucinations as the dog’s behavior suggested fear and an escape response. Owners of affected dogs stated that they were unable to walk the dog off the leash because if it occurred then the affected dog could run off uncharacteristically. As the disease progresses clinical signs become more intense and often dogs may be euthanized due to their severity. Unlike in humans, LD affected MWHD do not enter a vegetative state in constant myoclonus. This is likely a reflection that they are euthanatized before this stage but also because of their longevity. Affected humans develop the disease in late childhood to early adolescence and die at approximately 25 years of age. It is possible that the dogs do not develop more severe signs because they would die of natural causes first [1].

A genetic database of MWHD bred in the United Kingdom aimed at identifying affected and carrier animals was established by the Dachshund Breed Council [25]. The genetic test offered by the University of Toronto separates the normal, carriers and genetically affected dogs and is now an official DNA screening test recommended by the UK Kennel Club and a requirement for any MWHD breeder who wishes to be accredited in the “Assured Breeder Scheme” [27]. The genetic test is Southern blotting based [12], because PCR-based sequencing options through the massive dodecamer repeat expansion mutation have so far proven inadequately reliable. As such a larger amount of DNA is required than can be obtained from cheek swabs, and generally a blood sample is used. For this reason, the test is still not available through a commercial laboratory. The Southern blot based approach (Fig 2) is presently the only approved test but is expensive for the dog owner and is performed intermittently on batched samples which limits the number of dogs tested. In particular, clinically affected dogs presented to veterinary surgeons are less likely to be tested. Based on data published by the UK Kennel Club the frequency of carriers (34%) and genetically affected dogs (6%) is high [27] although this figure does not include hereditary clear dogs and the estimated carrier and affected rate may be closer to 20% (Ian Seath, personal communication). The genetic test is the definitive test in confirming the disease and the percentage for mutation positive dogs has decreased since breed wide testing has been implemented.

The precise roles of the *Epm2a* and *Epm2b* genes remain unknown. What is clear is that the former encodes a phosphatase (laforin) that regulates a small but important amount of phosphate covalently bound to glycogen. This phosphate appears to be critical to ensuring the sphericity of glycogen, which is essential to its solubility. *Epm2b*, the gene commonly mutated in dogs, encodes an ubiquitin E3 ligase (malin). Which proteins are the precise target(s) of malin remains unknown, but absence of malin’s function, like absence of laforin, leads to poorly branched and insoluble glycogen (polyglucosans), which accumulate in neuronal somatodendritic compartments into Lafora bodies (polyglucosan bodies), over time driving the neurodegeneration and progressive myoclonic epilepsy[28] (Fig 3).

Neuronal somatodendritic polyglucosan bodies in young animals or humans are essentially pathognomonic of LD. In human or canine LD, polyglcuosan bodies are also seen in various tissues outside the brain, such as muscle, heart and liver, but in these organs, they appear to be clinically neutral, usually the patient succumbing to the neurological disease without ever having developed clinical signs of hepatic, cardiac or skeletal muscle disease. Polyglucosan bodies can also be associated with other neurological syndromes often referred to in humans as polyglucosan body disease [29]. In these cases, the polyglucosan bodies are present not in the somatodendritic compartments of neurons, but in their axons or axon hillocks, leading not to an epileptic condition but to an axonopathy resembling amyotrophic lateral sclerosis. Similar syndromes occur in other species including dogs [30] cats [31], and the grey-head flying fox [32]. Non-pathogenic polyglucosan bodies occur as an aging-related change in many species including cats, dogs and man. These are usually present not in neurons but in glia, and in the human literature are termed corpora amylacea. They are an important consideration in canine LD, because in this species the disease is late in onset and brain necropsies could be misconstrued as representing LD in aged dogs [33–39].

Current therapy for LD is aimed at limiting clinical signs of the disease. However, all treatment is anecdotal as no research has been performed to assess the efficacy of individual therapies. Animals suffering from generalized seizures have received licensed anti-epileptic drugs such as phenobarbital at standard doses and unlicensed second or third generation anti-epilepsy drugs such as leviteracetam or zonisamide [9, 40]. Anecdotally, leviteracetam was found to be particularly effective in reducing myoclonus in early disease. In some cases where dogs suffer greatly from photosensitive myoclonus the use of canine specific sun goggles have been used to limit clinical signs. In human patients suffering from LD hallucinations occur regularly, causing anxiety. Although the presence of hallucinations in the canine disease can neither be confirmed nor denied, the use of anxiolyitcs, such as alprazolam, are potentially beneficial in dogs with LD.

Transgenically reducing brain glycogen synthesis by as little as 50% prevents the disease in LD mouse models and is an exciting potential therapeutic avenue [5, 21–23]. This is not possible to achieve merely through dietary restriction, e.g. by using low carbohydrate diets such as the ketogenic diet. Mice (our unpublished observation) and humans [41] in whom this has been tried maintain physiological blood glucose levels and generate just as profuse LB as with normal diet. Despite early promising results [42] longer term observations suggest that feeding a low CRcarbohydrate and/or high antioxidant diet does not delay onset of disease in LD dogs. Achieving reduced brain glycogen synthesis as a therapy for LD will therefore require approaches that directly downregulate glycogen synthesizing enzymes. In work recently published in abstract form, antisense oligonucleotides targeting glycogen synthase eliminated Lafora bodies in LD mouse models, establishing proof of principle for this strategy [43].

As demonstrated by our study, LD is a significant inherited condition in MWHD, and as such knowledge of the disease is pertinent in early detection and diagnosis. For any MWHD suffering with progressive myoclonus or seizure activity treatment with anti-epileptics should be initiated promptly. With the development of the genetic database it should become easier to identify the risk to each individual animal and eventually eliminate LD from the UK MWHD population. While providing a solid baseline for the progression of LD in MWHD this study suffers the weakness of being retrospective and owner based. To more comprehensively and definitively determine the natural progression and treatment of canine LD a prospective study looking at multiple animals with genetically confirmed disease should be completed. Work is very much underway to identify small molecule or other glycogen synthesis inhibitors to achieve 50% reduction in brain glycogen synthesis. A detailed canine natural progression study will allow organization of clinical trials also in dogs. It must be emphasized that dogs with LD, even in the MWHD breed alone, outnumber all known cases of human LD. All affected dogs to date, from all breeds, have the same expansion mutation in the *Epm2b* dodecamer repeat unique to the canid orthologue of the gene. LD, in this sense, can be regarded as a canine genome particular disease, and is likely to continue to be seen in different purebred breeds with each recurrence of the expansion mutation.

## Supporting information

S1 Text Survey for owners of LD affected dogs. To improve survey accuracy technical terms were explained within the survey for example generalized seizures were explained as “also known as grand mal or tonic clonic and characterized by initial stiffening of the limbs followed by jerking of the limbs and face”. The survey participant was asked to enter the dog’s age that they first noticed the clinical signs or “never” if this sign was not noticed. There were more specific questions about urinary incontinence to enable assessment as to whether incontinence occurred as a result of loss of house training (i.e. disinhibition and a specific sign of senility) or was more likely due to other causes. There was also open questions to allow participants to enter other clinical signs and other comments. Participants were also asked their opinion on the impact of LD on daily living. Finally there was a question about possible comorbidities including previous diagnosis of intervertebral disease. This is the most common neurological disease in this breed which could be alternative cause of the clinical sign of ataxia.
(PDF)

S1 Table Raw data received from LD survey in microsoft excel. (XLSX)

S1 Movie Video of LD affected dogs. Daisy a 13 year old female spayed MWHD together with her 10 year male neutered son Bertie. Both are mutation positive for LD. As an older dog Daisy is more severely affected and is nearly blind and has a high stepping hypermetric gait particularly of the pelvic limbs with a pelvic limb tremor. Both dogs have spontaneous and reflex myoclonic jerks with an intention tremor.
(MP4)
