# Supplementary material for: Lafora disease in miniature Wirehaired Dachshunds
Source: PLoS One. 2017 Aug 2;12(8):e0182024. doi: 10.1371/journal.pone.0182024 (PMC5540395; doi:10.1371/journal.pone.0182024)
Supplement: S1 Text — To improve survey accuracy technical terms were explained within the survey for example generalized seizures were explained as “also known as grand mal or tonic clonic and characterized by initial stiffening of the limbs followed by jerking of the limbs and face”. The survey participant was asked to enter the dog’s age that they first noticed the clinical signs or “never” if this sign was not noticed. There were more specific questions about urinary incontinence to enable assessment as to whether incontinence occurred as a result of loss of house training (i.e. disinhibition and a specific sign of senility) or was more likely due to other causes. There was also open questions to allow participants to enter other clinical signs and other comments. Participants were also asked their opinion on the impact of LD on daily living. Finally there was a question about possible comorbidities including previous diagnosis of intervertebral disease. This is the most common neurological disease in this breed which could be alternative cause of the clinical sign of ataxia. (PDF) [file pone.0182024.s001.pdf]

Edit this form

Lafora Disease Progression Survey

This survey is part of a project to research the progression of Lafora Disease in Miniature Wire-haired Dachshunds. It is supported by the Dachshund Breed Council's Health Sub-committee and Dr. Clare Rusbridge (Veterinary Neurologist).

We would really appreciate the cooperation once again from any owners of dogs that are showing clinical signs of Lafora, or that have been tested as genetically affected, even if they are not yet showing any symptoms. This is a vital part of monitoring the possible impact of the condition over time. All data collected will be kept confidential and only used for research purposes. No owners' names, or dogs' names will be published. By completing this survey you consent to having your data used by this research project.

Questions marked with an asterisk (*) are mandatory. All other questions are optional. IF YOU HAVE COMPLETED THE FORM BEFORE, THE ONLY ESSENTIALS ARE YOUR NAME, YOUR DOG'S NAME AND ANY NEW SYMPTOMS IN THE LAST 12 MONTHS, BUT FEEL FREE TO COMPLETE ANY OTHER QUESTIONS IF YOU WISH.

If you have more than one dog that is, or may be, affected by Lafora, please complete additional surveys, using the same survey web address (you will be given the option to submit an additional survey at the end of this one).

Please ONLY complete this survey if you are a UK owner of a Miniature Wirehaired Dachshund.

* Required

You and Your Dog

Your first name *

Your Surname *

Please confirm your e-mail address. *
your_e-mail@domain_name.co.uk

Your dog's Pet Name. *

Your dog's Kennel Club Registered Name, if known.

Is your Dachshund a dog or a bitch? *

- Dog
- Bitch

What is the current sexual status of your dog? *

- Neutered
- Not Neutered

If your dog is a bitch, has it ever had a litter of puppies and if so how many?

- Never
- Yes - 1 litter
- Yes - 2 litters or more
- Not a bitch

Sorry to ask this, but In what month and year did your dog die, if it has? (mm/yy)
Ignore this question if your dog is still alive.

Has your dog been DNA tested using the WHDC screening programme as being "Affected" by Lafora Disease? *
The WHDC has been screening dogs with a DNA test since 2010.

- Yes
- No
- Other:

Continue »

33% completed

Powered by
Google Forms

This content is neither created nor endorsed by Google.

Google Forms
Report Abuse - Terms of Service - Additional Terms

Edit this form

Lafora Disease Progression Survey

* Required

Lafora Disease Symptoms

IMPORTANT NOTE:

IF YOU HAVE COMPLETED THE SURVEY BEFORE, PLEASE ANSWER THE FIRST QUESTION BUT ONLY REPORT ANY ADDITIONAL SYMPTOMS IN THE LAST 12 MONTHS - WE HAVE YOUR PREVIOUS ANSWERS ON RECORD, THANK YOU.

Has your dog ever shown any symptoms that might be related to Lafora? *

If 'no', please skip to the 'Other Information' section

- ◯ Yes
- ◯ No

Has your dog been clinically diagnosed, by a veterinary surgeon, as having Lafora Disease? *

- ◯ Yes
- ◯ No

At what age did symptoms first start?

(Age in years, to the nearest half year; e.g. 5.5)

When the symptoms first started, did your Vet recognise them as being possibly the result of Lafora?

Please answer for the original vet if you subsequently had a second opinion.

- ◯ Yes
- ◯ No

What signs prompted you to take your dog to the vet?

What did your vet suggest that the symptoms may be due to / what did your vet diagnose?

Did your vet suggest a referral to a neurology specialist?

Think back to your answer above ‘at what age did symptoms first start?’. Have you seen any of the following symptoms which may be connected with Lafora, and if so when did they first appear? .

The drop-down menu gives you a set of choices from "Never" to "Age 15 or older". (e.g. d you saw partial seizures at age 9)

Myoclonic jerking/twitching - reaction to stimulus such as light

The drop-down menu gives you a set of choices from "Never" to "Age 15 or older". e.g. you saw jerking for the first time age 5

Myoclonic jerking/twitching - when sleeping or falling asleep (hypnic jerks)

The dropl-down menu gives you a set of choices from "Never" to "Age 15 or older". e.g. you saw partial seizures for the first time age 7

Generalised seizures

Also known as grand mal, tonic clonic and characterised by initial stiffening of the limbs (the tonic phase), followed by jerking of the limbs and face (the clonic phase). The dog may vomit, drool, urinate or defecate. What age did you first see this, if at all

Partial seizures

Also known as focal and characterised by seizure activity that only involves part of the brain. The dog may show focal twitching or movement and/ or have reduced awareness or abnormal behaviour. What age did you see this, if at all

Fly catching

(Look as if catching flies i.e. focus on an "object" and snap - even leaping for "it")

Jaw smacking/chewing

(Can be seen in generalised / partial seizures)

Panic attack

Aggression to other dogs

Aggression to people

Blindness/poor eyesight

Deafness/hearing problems

Dementia

Incontinence - urinary

Incontinence - fecal

Incontinence - please tell us which, if any, of these options applies to your dog

(Tick as many as are relevant)

- ☐ Eliminates indoors (lost house training)
- ☐ Dribbles urine without being aware
- ☐ Drops faeces without being aware
- ☐ Urinates during seizures
- ☐ Defecates during seizures

Other symptoms you have noticed

Symptoms you feel may be associated with Lafora, e.g. periodic stiffness in muscles

Description of symptoms

If you would like to give us a text description of your dog's initial and/or current symptoms, please do so here.

« Back Continue »

66% completed

Powered by Google Forms

This content is neither created nor endorsed by Google.

Report Abuse - Terms of Service - Additional Terms

Edit this form

Lafora Disease Progression Survey

* Required

Other Information

Has your dog ever been diagnosed with Pancreatitis?

Several owners have mentioned that their dogs have suffered from pancreatitis. We hope to establish whether or not Lafora affected dogs are more prone to the disease than the general population.

- Yes
- No

If you answered "Yes" to the previous question, how old was your dog when it was diagnosed with Pancreatitis?

(Age in years to the nearest half year; e.g. 5.5)

If your dog has been diagnosed with Pancreatitis please tell us about the age of diagnosis and any other information you feel may be helpful.

(Please tick all that apply, from the following list)

Thinking about the present only, (or the month before the point of death), please record your opinion on the general impact of Lafora on daily living for both your dog and yourself.

- No symptoms, so none
- Slight symptoms but no impact
- Some impact (e.g. different diet, slight change walking patterns etc.)
- Significant impact (e.g. difficulty walking, severe fits, etc. but day to day life is manageable .)
- Life changing impact (e.g. can't travel, walk etc. easily, limiting what you can do)

○ So severe considering putting dog to sleep.

What advice would you give to other owners, based on your experiences? (AGAIN, IF YOU HAVE ANSWERED BEFORE, DON'T FEEL OBLIGED TO COMPLETE THIS QUESTION)

Any further information

Please feel free to add any more information to explain your answers.

Please confirm whether you are prepared to help with our continuing research, by repeating this survey in 6 months (or if, sadly, your dog should die)? *

This survey is part of a progressive survey to track Lafora symptoms as they develop over time. Your continuing support is appreciated.

○ Yes

○ No

Thank you for completing our survey into the progression of Lafora Disease in Mini Wire Dachshunds.

If you have more than 1 dog that is, or may be, affected by Lafora, please complete additional surveys, using the same survey web address (you will be given the option to submit an additional survey at the end of this one).

All data collected will be kept confidential and only used for research purposes. No owners' names, or dogs' names will be published. By completing this survey you consent to having your data used by this research project.

« Back | Submit

100%: You made it.

Never submit passwords through Google Forms.

Powered by
Google Forms

This content is neither created nor endorsed by Google.

Report Abuse - Terms of Service - Additional Terms
